# Gossip, Rumors, and the COVID-19 Crisis

**DOI:** 10.1017/dmp.2020.272

**Published:** 2020-07-27

**Authors:** Amir Abdoli

**Affiliations:** Zoonoses Research Center and Department of Parasitology and Mycology, Jahrom University of Medical Sciences, Jahrom, Iran

**Keywords:** COVID-19, gossip, rumor, SARS-CoV2, social media

On March 11, 2020, the World Health Organization announced that the coronavirus disease (COVID-19) is a pandemic crisis. Besides the disease itself, the fear from COVID-19 has a high psychological burden (eg, psychological distress, anxiety, and depression) on patients and their families, medical staff, health care workers, and the general population.^1^ These days, we are faced with a lot of information regarding COVID-19 from different sources. However, fake information, gossip, and rumors seem to be more common than factual information about this disease.

Notwithstanding the advantages of social media, unfortunately, they are also a major source of misinformation. For instance, some peoples have believed that the severe acute respiratory syndrome coronavirus 2 (SARS-CoV-2), the virus responsible for causing COVID-19, is transmitted through the 5G networks, and, consequently, some of the 5G network towers were destroyed or damaged due to this fake news.^2^ The effectiveness of traditional medicine against COVID-19 is another common rumor, as well as the consumption of certain foods and beverages (eg, lukewarm water, alcohol, garlic, onion, ginger, and sea lettuce), which has been reported to be effective against COVID-19. Furthermore, strange methods, including the application of sesame oil into the body, granite baths, and smoking harmala have been recommended for the prevention of COVID-19.^3^ While the COVID-19 vaccine could be a promising strategy for preventing the infection, anti-vaccine activists are already commenting on this topic on Twitter and Facebook, even before an approved vaccine becomes available.^4^ As reported by *Science* magazine, only about 50% of Americans plan to get the COVID-19 vaccine, and, in Europe, only 26% in France reported that they will not get the vaccine.^4^ Exaggerated information about the case fatality rate and pandemic estimates is another challenge of this crisis hinted at in social media.^5^ It is observed that social media exposure (SME) has been associated with anxiety and depression among the users.^6^ A recent study during the COVID-19 outbreak in China has shown that SME was positively associated with anxiety (odds ratio = 1.72) and combined depression and anxiety (odds ratio = 1.91) among the Chinese population.^6^ Increasing pandemic fears of COVID-19 due to misinformation is another disadvantage of the social media platforms.^5^

Gossip and rumors are not only diminishing the mental health status,^6^ but also may be interfering with the processes of diagnosis, prevention, and treatment of COVID-19. Therefore, planning strategies for coping with fake news during this crisis should be considered by the government and the health authorities. It is suggested that more research will be conducted to identify the association of social media and COVID-19-related stress. Also, it is recommended that health authorities be more active in social media, especially during the outbreak of diseases to disseminate accurate information and factual news.
